# Editorial Notes, News and Miscellany

**Published:** 1913-08

**Authors:** 


### Editorial Notes, News and Miscellany.

Dear Doctor.—I herewith hand you my check for subscription to your most interesting and educational journal, "Red Back." You are doing a great work in discussing the law of heredity and instructing the doctors on the principles of eugenics. Keep up the noble work you are doing, and you will receive your reward.

---

Contagion.—Little George had heard a great deal said about disease germs, such as tuberculosis, etc. One day the family were at dinner, and George wanted a drink of water. The tired mother said:

"Drink out of your uncle's glass, George; he is through eating."

The little fellow commenced to cry, and said:

"I don't want to; I'm afraid I will catch the backache."—*Eustis Lake Region.*

---

Review Retreat—a pretty name—is what Dr. C. L. Gregory call his sanitarium for nervous diseases, alcoholism and drug addiction, which he has just opened at Greenville, Texas. Dr. Gregory has had unusual opportunities for studying and treating this class of patients. He was formerly Superintendent of North Texas Hospital for the Insane at Terrell, Texas, during which time he had more than six thousand patients under his charge. The Superintendent and his family live in the Sanitarium, which makes it home-like in its management, and enables him to give every patient his personal attention.

---

When it is Hot.—Consider Mr. Shadrach, of fiery furnace fame: he didn't bleat about the heat or fuss about the flame. He didn't stew and worry, and get his nerves in kinks, nor fill his skin with limes and gin and other "cooling drinks." Consider Mr. Meshach, who felt the furnace, too: he let it sizz, nor queried "Is it hot enough for you?" He didn't mop his forehead, and hunt a shady spot; nor did he say, "Gee! what a day! Believe me, it's some hot!" Consider, too, Abed-nego, who shared his comrades' plight: he didn't shake his coat and make himself a holy sight. He didn't wear suspenders without a coat and vest; nor did he scowl and snort and howl and make himself a pest. Consider, friends, this trio—how little fuss they made. They didn't curse when it was worse than ninety in the shade. They moved about serenely within the furnace bright, and soon forgot that it was hot, with "no relief in sight."—*Chicago Tribune.*

